# Primary extranodal NK/T cell lymphoma, nasal-type of uterus with adenomyosis: a case report

**DOI:** 10.1186/1746-1596-9-95

**Published:** 2014-05-23

**Authors:** Jian-chen Fang, Jue Zhou, Zheng Li, Zhao-xia Xia

**Affiliations:** 1 Ningbo Diagnostic Pathology Center, Ningbo 315031, China

**Keywords:** Uterus, NK/T cell lymphoma, Extranasal type

## Abstract

Natural killer (NK)/T cell lymphoma of the female genital tract is extremely rare. We here report a case of ‘nasal type’ NK/T cell lymphoma arising in the uterus with adenomyosis in a 41-year-old woman with fever and hypogastralgia. The histologic analysis demonstrated a highly aggressive tumor with characteristic angiocentric/angiodestructive growth pattern and focal necrosis. The lymphoma cells displayed a CD3ϵ/CD56/TIA-1/granzyme-B/Perforin-positive and CD20/CD79a/CD4/CD8-negative immunophenotype and positive for Epstein-Barr virus by EBER in situ hybridization. Clinically, the disease was limited to the uterus at the initial diagnosis, but progressed rapidly. The patient died on day 54 after hysterectomy, irrespective of intensive chemotherapy.

**Virtual Slides:**

The virtual slide(s) for this article can be found here: http://www.diagnosticpathology.diagnomx.eu/vs/1323474831125945

## Background

Primary lymphoma of the female genital tract is uncommon with a frequency of only 0.002% in all patients with extranodal lymphomas [1]. The majority of these cases represent aggressive B-cell lymphomas. Involvement of the gynecological tract by NK/T cell lymphomas is considered to be extremely rare and only 5 cases with primary NK/T cell lymphoma involving the endometrium of the uterus has been reported in the English literature [2-5]. There was no report of such case in uterus associated with adenomyosis. Here, we report the first case of primary NK/T cell lymphoma arising in the uterus with adenomyosis.

## Case presentation

### Clinical history

A 41 year-old woman, without relevant previous anamnesis, presented with fever and hypogastralgia for 2 months. Computer tomography and ultrasonography revealed enlargement of the uterus and multinodular intrauterine mass. A hysterectomy was performed. She was diagnosed as extranodal NK/T cell lymphoma, nasal-type. After surgical resection, the patient was treated with CHOP chemotherapy (cyclophosphamide, vincristine, daunorubicin and dexamethasone). Despite extensive chemotherapy, the disease progressed rapidly; shortly follow-up radiological imaging showed the retroperitoneal involvement. The patient died on day 54 after surgical resection of the tumor.

### Pathological findings

**Figure 1:**
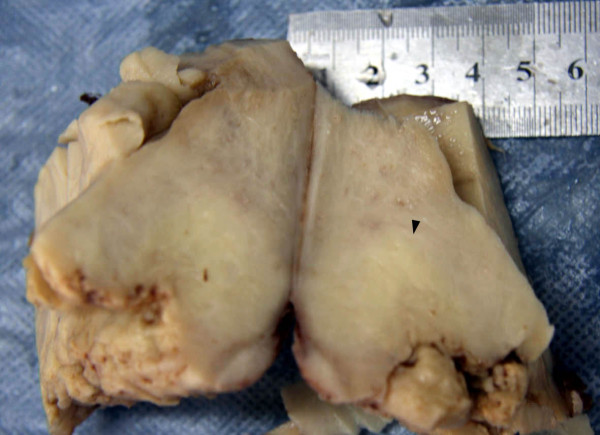
**Macroscopic view.** Macroscopic examination displayed a yellow, soft, poorly circumscribed mass.

**Figure 2:**
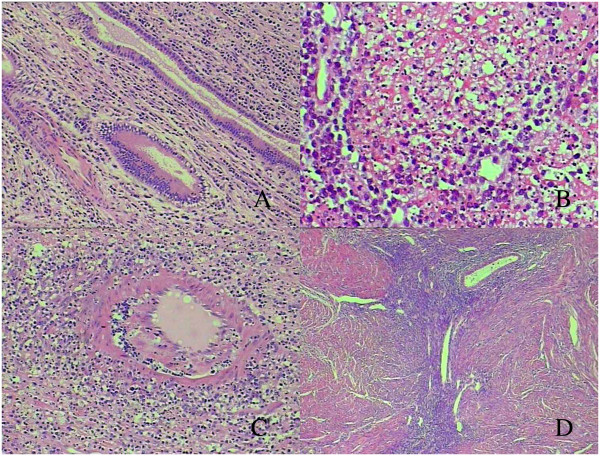
**Histological features of this case. A**, Dense lymphomatous cells infiltrate endometrium,partially covered by intact columnar epithelium (HE, ×40 magnification). **B**, The lymphomatous proliferation was interrupted by coagulative necrotic areas (HE, ×200 magnification). **C**, Angiocentric and angiodestructive growth pattern is frequently present (HE, ×100 magnification). **D**, Adenomyosis in myometrium without lymphomatous infiltrate (HE, ×40 magnification).

Macroscopic examination displayed a yellow, soft, poorly circumscribed mass that invaded about 4 cm in uterine wall (Figure 1). Histopathological evaluation revealed lymphomatous infiltrate the endometrial gland (Figure 2A) and myometrium with well demarcated large areas of coagulative necrosis containing apoptotic nuclear debris (Figure 2B). The tumor cells demonstrated a prominent angioinfiltrative growth pattern with concentric arrangement around small arteries (Figure 2C). The lymphoma cells were densely packed, with an abundant cytoplasm and enlarged nuclei with open chromatin and several large nucleoli. Mitotic figures were frequently seen. There was adenomyosis in myometrium without tumour involvement (Figure 2D).

**Figure 3:**
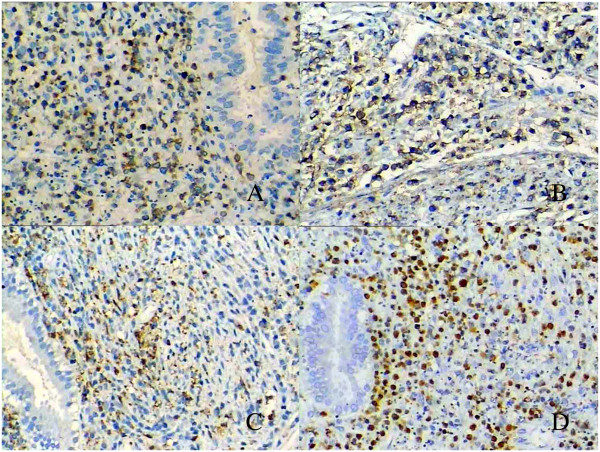
**Phenotypic characteristics of tumor cells. A**, The lymphoma cells showed cytoplasmic CD3ϵ **B** and **C**, Membranous CD56 and cytoplasmic granular TIA-1 positivity by immunohistochemistry (×200 magnification). **D**, In situ hybridization for EBER sequences clearly supported EBV infection of the lymphoma cells (×200 magnification).

The tumor cells were positive for cytoplasmic CD3 and membranous CD56 (Figiures 3A and Figure 3B) but negative for CD4, CD5, CD8, CD20, CD79α, CD30. Cytotoxic proteins TIA-1 (Figure 3C), granzyme-B and Perforin displayed strong cytoplasmic granular staining pattern. EBER in situ hybridization demonstrated strong positivities for all tumor cells (Figure 3D). Based on the overall morphological, immunophenotypical and EBV characteristics, the diagnosis of extranodal (uterine) NK-cell lymphoma, nasal-type was made.

## Discussion

Extranodal NK/T-cell lymphomas characteristically involve the upper aerodigestive tract, with the nasal cavity being the prototypic site [6]. Rarely, the tumour occurs in prostate, adrenal glands and lung [7-9]. Thus far, only a few cases of T- or NK/T-cell neoplasms involved uterus have been reported [2-5]. Like most of NK/T cell lymphoma in other anatomic sites, these lymphomas in the uterus usually are highly aggressive, and conventional prognostic factors usually fail to predict their outcome [10]. While clinical presentation of NK/T cell lymphoma involving the uterus may include vaginal bleeding, and abdominal or pelvic pain [11], our patient presented with fever and hypogastralgia with no evidence of vaginal bleeding. As the common pathological features of NK/T cell lymphoma defined by WHO classification, the current case demonstrated a highly aggressive tumor with characteristic angiocentric/angiodestructive growth pattern and associated focal necrosis.

Interestingly, there is uterus adenomyosis present nearby the lymphoma in the current case; perhaps it might be an important factor contributing to the onset and process of the tumor. Occurrence of T cells and CD56+ NK cells within the normal endometrium has been described [12]. It seems to be established that uterine NK cells form a dynamic lymphoid pool in each menstruation cycle. One could expect that these cells may frequently undergo genetic and regulatory errors leading to malignant transformation [2]. For this reason it is difficult to understand, why uterine NK-cells transform to malignant lymphoma with such a low frequency. As one possible explanation, the relatively short duration of a menstrual cycle and the regular shed of the endometrium may prevent the expansion and malignant transformation of NK cells [2]. In the presented case, the presence of adenomyosis may prevent the normal NK cell duration and cycling in the uterus, perhaps provide the evidence that NK cells frequently remained in uterine corps undergo genetic and regulatory errors leading to malignant transformation. The association of adenomyosis with NK/T-cell lymphoma was largely not mentioned previously, because that the majority of cases reported with NK/T cell lymphoma in uterus often diagnosed by curettage, the way impossible to find adenomyosis.

According the WHO criterion, the neoplastic lymphoid cells usually coexpress NK cell markers such as CD56 and T cell-associated antigens like CD3, CD2 with expression of cytotoxic markers such as TIA-1, Perforin and Granzyme-B [6]. The current case demonstrated the immuno characteristics of tumor cells are typical nasal-type NK/T cell lymphoma: CD3+, CD56+, TIA1+, Perforin+, Granzyme B+. As a hallmark of nasal type NK/T cell lymphoma, EBV in situ hybridization clearly supported EBV infection of the lymphoma cells. TIA-1 and EBER were the two most sensitive markers of the disease. However PCR-based TCR gene rearrangement analysis might not be a useful technique for making diagnosis of NK/T cell lymphoma [13]. Latent membrane protein (LMP) 1 and LMP2A encoded by Epstein-Barr virus were associated with the development of malignancies. High expression of the two proteins could independently predict poor overall survival [14].

NK/T cell lymphomas were reported to have a median survival of only 0.28 years [15]. One case of NK/T cell lymphomas involved prostate was reported recently, the patient died within 4 months after diagnosis [7]. In another case of the lymphomas occurred in bilateral adrenal glands, the patient died only 33 days after initial presentation [8]. In the present case, the patient died 54 days (0.15 year) after hysterectomy. Unfortunately, treatment experience is mostly limited to the upper aerodigestive tract disease. Extranodal NK/T cell lymphomas of other sites are extremely rare and very limited data for optimal treatment strategies are currently available.

## Conclusion

This case demonstrated a rare NK/T cell lymphoma primarily in the uterus, providing a diagnostic pitfall: pathologists and gynecologists should be aware of its existence and need to consider NK/T cell lymphomas within the spectrum of differential diagnosis of neoplastic tumor in the uterus. Because of the clinical aggressiveness and dismal prognosis of the tumor, more effective therapeutic regimens should be actively looked.

## Consent

Written informed consent was obtained from the family of the patient for publication of this case report and any accompanying images. A copy of the written consent is available for review by the Editor-in-Chief of this journal.

## Competing interests

The authors declare that they have no competing interests.

## Authors’ contributions

JC F analyzed the data and wrote the manuscript as a major contributor. ZX X, Z L helped to perform the immunochemical staining. J Z helped to revise the discussion section of this manuscript. All authors have read and approved the final manuscript.
